# Small Dense Low-Density Lipoprotein Level in Newly Diagnosed Type 2 Diabetes Mellitus Patients With Normal Low-Density Lipoprotein

**DOI:** 10.7759/cureus.33924

**Published:** 2023-01-18

**Authors:** Ayesha Juhi, Kamlesh Jha, Himel Mondal

**Affiliations:** 1 Physiology, All India Institute of Medical Sciences, Deoghar, IND; 2 Physiology, All India Institute of Medical Sciences, Patna, IND

**Keywords:** small dense low-density lipoprotein, low-density lipoprotein, ldl cholesterol, serum lipid profile, atherosclerosis, lipoproteins, diabetes mellitus, cardiovascular diseases, cholesterol

## Abstract

Background and objective

There are three subtypes of low-density lipoprotein (LDL): large buoyant (lb), intermediate, and small dense (sd). Among these LDL subtypes, small dense low-density lipoprotein (sdLDL) has been proven to be an independent risk factor for atherosclerosis. Type 2 diabetes mellitus (T2DM) encompasses several metabolic abnormalities and patients suffering from T2DM without good glycemic control are prone to develop atherosclerosis. Hence, in T2DM, it is recommended to evaluate blood lipids for early detection of hyperlipidemia to identify the risk. A larger percentage of sdLDL in T2DM patients with an optimal or near-optimal LDL level might be a hidden risk factor for atherosclerosis. Hence, we aimed to find the level of sdLDL cholesterol (sdLDL-C) among newly diagnosed T2DM patients with optimal or near-optimal blood lipids and to compare it with age and sex-matched controls.

Materials and methods

In this study, we enrolled newly diagnosed T2DM patients from the diabetic clinic of a tertiary care hospital. The patients were then tested for blood lipids, namely, total cholesterol, triglyceride, high-density lipoprotein cholesterol, and low-density lipoprotein cholesterol (LDL-C), including sdLDL-C. Then, age and sex-matched controls with similar baseline lipid levels to the T2DM group (without sdLDL-C) were recruited. After recruitment, both groups were measured for blood lipids including sdLDL-C in a single day. The level of sdLDL-C between the groups was tested statistically by the Mann-Whitney U test.

Results

A total of 50 T2DM patients with a median age of 36 years (Q1-Q3: 33.75-41) were included as the study group and 50 age and sex-matched controls with a median age of 34 years (32-37.25; p = 0.09) were recruited. The median fasting glucose was 165 (Q1-Q3: 145-199.25) mg/dL and 90.5 (Q1-Q3: 87.75-95.25) mg/dL (p < 0.0001) in the study and control groups, respectively. The LDL-C was 109.9 (Q1-Q3: 99.4-119.4) mg/dL and 108.5 (Q1-Q3: 87.55-124.1) mg/dL (p = 0.94) in the study and control groups, respectively. The sdLDL-C was 40.11 (Q1-Q3: 36.28-43.58) mg/dL and 24.64 (Q1-Q3: 22-32.49) mg/dL (p < 0.0001) in the study and control groups, respectively.

Conclusion

Newly diagnosed T2DM patients with blood lipids within an optimum or near-optimum level may have a higher percentage of sdLDL-C when compared with healthy controls. Hence, they may have a higher risk of atherosclerosis and cardiovascular diseases. Clinicians may miss the potential risks if they do not advise the sdLDL-C component of LDL-C while advising for the test for blood lipid.

## Introduction

The chronic consequence of type 2 diabetes mellitus (T2DM) is accelerated coronary, cerebral, and peripheral vascular atherosclerosis. When compared to people without diabetes, the death rate from coronary heart disease (CHD) is up to four times greater. Also, major adverse cardiovascular events, apart from mortality, greatly increase healthcare costs [1]. Dyslipidemia is one of the causes that increase the risk of cardiovascular disease due to a higher propensity to atherosclerosis. Low-density lipoprotein (LDL) raises the risk of atherosclerosis and consequent cardiovascular illnesses, whereas high-density lipoprotein (HDL) has a cardioprotective effect [2].

LDL has multiple subclasses according to size, density, chemical, and metabolic activity and these properties are responsible for the difference in their atherogenicity. The size and stickiness of large buoyant (lb), intermediate, and small dense (sd) LDL are different [3]. Compared to large buoyant LDL (lbLDL), small dense LDL (sdLDL) is significantly smaller and stickier. As a result, in circulation, sdLDL is more prone to become stuck inside the arterial wall [4]. In addition, the lbLDL particles are quickly removed and recycled by the liver. However, sdLDL does not fit well on the LDL receptors in the liver. Hence, their presence is more in circulation.

Circulating sdLDL is prone to oxidation, glycation, and desialylation. These modifications heighten atherogenicity. There is a higher affinity of the sdLDL to heparin sulfate proteoglycans in the vessel wall and this increases the penetrability of sdLDL, and after penetration, they enhance the formation of foam cells [5].

Newly diagnosed T2DM patients are commonly screened for the lipid profile and antilipidemic drugs are suggested if the levels are high for the individual [6]. Those who have their LDL cholesterol (LDL-C) in the normal range may have underlying risks of cardiovascular diseases associated with a higher percentage of sdLDL cholesterol (sdLDL-C) level.

In this context, we designed this study to find out the level of sdLDL-C in newly diagnosed T2DM patients who are having a normal level of LDL-C. The outcome would help us to know the pattern of sdLDL-C in those patients considered apparently normal according to the level of LDL-C.

## Materials and methods

Type and settings

This was a cross-sectional observational study where variables were measured in a study group and a control group and compared. Research participants were recruited from the diabetes clinic of a tertiary care hospital. The study was conducted from January 2019 to December 2020.

Ethics

We recruited only adult (age > 18 years) patients for this study. All the patients were approached with a brief description of the study aim and procedure and recruited after they voluntarily consented to participate. The Institutional Ethics Committee of Apollo Medical College and Hospital, Hyderabad, India approved this study (AIMSR/IRB/EC/2018/10/075).

Minimum sample size

For the calculation of sample size, we used our expectations with results from a previous study [7]. We expected an sdLDL-C in the study and control groups at 44 mg/dL and 34 mg/dL, respectively, with an expected standard deviation of 10. Along with these data, the alpha was set at 0.05, and the beta was set at 0.1, which indicates the power of the study was 90%. The calculated minimum sample size was 42 in two groups. However, for a higher power of the study, we aimed to include 50 patients in each group.

Recruitment process

**Figure 1:**
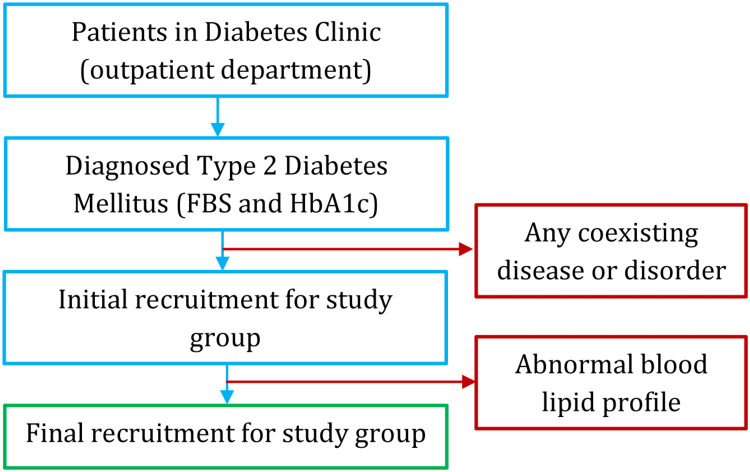
Recruitment of the research participants in the study group FBS: fasting blood sugar; HbA1c: glycated hemoglobin.

Patients who were diagnosed with T2DM by fasting blood sugar (FBS) and glycated hemoglobin (HbA1c) measured on two occasions were approached for a detailed history and co-existing disease or ongoing treatment. A thorough clinical examination was conducted by an expert clinician. Those with no apparent co-existing disease or disorder were recruited and tested for blood lipid profile. Participants with an abnormal level of lipids were excluded to get the final pool of research participants. The recruitment process is shown in Figure 1.

After recruiting the study group, a control group of similar age and sex was recruited from the same tertiary care hospital from the patient’s attendants. They also went through the same procedure of history, clinical examination, and blood tests as the study group.

Criteria for inclusion

The diagnosis of T2DM was based on the measurement of fasting blood glucose and HbA1c. If the blood glucose was above 125 mg/dL and HbA1c was above 6.5% on two occasions after a 15 days gap, the patient was diagnosed to be suffering from T2DM [8]. After the recruitment of T2DM patients, they underwent a blood test for LDL-C. None of them were on lipid-lowering drugs. Those who had a level of LDL-C ≤ 129 mg/dL (optimal < 100 mg/dL and near-optimal range: 100-129 mg/dL) were finally included in the study group [9]. We did not use any other criteria based on the level of triglycerides, total cholesterol, or HDL cholesterol (HDL-C) for the recruitment of the participants.

Blood test

A venous blood sample was collected from the antecubital vein maintaining aseptic precautions after 12-hour fasting. The FBS, HbA1c, and lipid profile were again tested and this result was stored for analysis. The sdLDL-C was measured by quantitative Denka Seiken (Tokyo, Japan) assay that quantifies sdLDL-C in blood samples. The test uses a two-step procedure to quantify sdLDL-C. In the first step, using a surfactant and sphingomyelinase, chylomicrons and lipoproteins (very low, intermediate, and low density) are removed. In the second step, a surfactant releases cholesterol solely from sdLDL-C so that it can be measured using conventional techniques [10].

Anthropometry

The research participants were measured for weight, height, and body fat percentage (bioelectrical impedance method of measurement), and body mass index was calculated from the collected data. Single clinicians with previous expertise in anthropometry measured all the parameters. For the measurements of women, a women's attendant was present and prepared the subjects for height and weight measurement. A portable stadiometer (Precision Model, PrimeSurgicals, New Delhi, India) was used to measure the height to the nearest 1 mm. The weight was measured concurrently with the body fat percentage with the Omron Body Composition Monitor HBF-375 (Omron Corporation, Kyoto, Japan). The precautions for body fat measurements were followed before the body fat estimation [11].

Statistical analysis

Data were checked for distribution by the Shapiro-Wilk test and found not to follow the normal distribution. Hence, data were presented as median and first and third quartile (Q1-Q3) [12]. Mann-Whitney U test was used for comparing the medians in the study and control groups. We used GraphPad Prism 6.01 (GraphPad Software, San Diego, CA) for the statistical tests. A p-value < 0.05 was taken as statistical significance.

## Results

A total of 50 T2DM patients with a median age of 36 years were included as the study group and 50 age- and sex-matched patients with a median age of 34 years were recruited as control. The age, sex, anthropometric parameters, FBS, and HbA1c level in the study and control groups are shown in Table 1.

**Table 1:** Age, anthropometric parameters, and fasting blood glucose levels in the study and control groups * Statistically significant p-value; ^†^ p-value is of the binomial test. BMI: body mass index; FBS: fasting blood sugar; HbA1c: glycated hemoglobin.

Variable	Study (n = 50)	Control (n = 50)	P-value
	Median (first quartile-third quartile)		
Age (years)	36 (33.75-41)	34 (32-37.25)	0.09
Sex ratio (male/female)	28/22	28/22	0.48^†^
Height (cm)	168.5 (163.875-175)	169.25 (163.88-175)	0.87
Weight (kg)	72.6 (65.7-81.6)	70.6 (64.5-81.2)	0.37
BMI (kg/m^2^)	26.35 (24.12-28.12)	24.97 (22.72-27.47)	0.21
Body fat (%)	33 (25.95-37)	33.7 (30.8-37.8)	0.15
FBS (mg/dL)	165 (145-199.25)	90.5 (87.75-95.25)	<0.0001*
HbA1c (%)	8.5 (7.95-9)	4.5 (4.2-5.53)	<0.0001*

The blood lipid profile including sdLDL-C in the study and control groups is shown in Table 2.

**Table 2:** Blood lipid parameters in the study and control groups * Statistically significant p-value. HDL-C: high-density lipoprotein cholesterol; LDL-C: low-density lipoprotein cholesterol; sdLDL-C: small dense low-density lipoprotein cholesterol.

Variable	Study (n = 50)	Control (n = 50)	P-value
	Median (first quartile-third quartile)		
Total cholesterol (mg/dL)	174.5 (165.75-185.75)	173 (161-188.25)	0.95
Triglyceride (mg/dL)	105 (93-105.25)	104 (94-145)	0.65
HDL-C (mg/dL)	42 (39-45)	44 (39-46)	0.27
LDL-C (mg/dL)	109.9 (99.4-119.4)	108.5 (87.55-124.1)	0.94
sdLDL-C (mg/dL)	40.11 (36.28-43.58)	24.64 (22-32.49)	<0.0001*

Despite having a similar level of LDL-C (median of 109.9 mg/dL in the study group versus 108.5 mg/dL in the control group, p = 0.94), the sdLDL-C was significantly higher (median of 40.11 mg/dL in the study group versus 24.64 mg/dL in the control group, p < 0.0001) among newly diagnosed T2DM patients.

## Discussion

**Figure 2:**
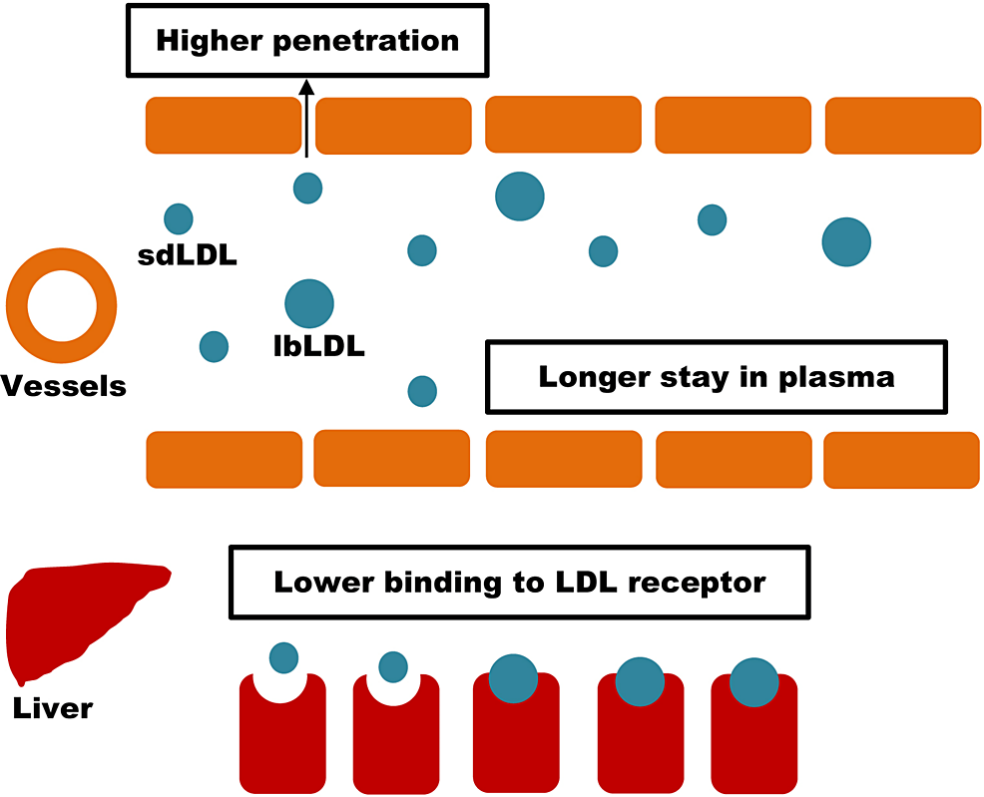
Comparative atherogenicity of small dense low-density lipoprotein and large buoyant low-density lipoprotein LDL: low-density lipoprotein; sdLDL: small dense low-density lipoprotein; lbLDL: large buoyant low-density lipoprotein. This figure was made by the corresponding author after reviewing references [13-15].

We found that despite having a similar level of LDL-C, patients diagnosed with T2DM had more percentage of sdLDL-C. The underlying reason for this finding may be due to the higher activity of hepatic lipase in a hyperglycemic state [13]. The hepatic lipase acts on the LDL and removes the triglycerides and forms sdLDL. The activity of hepatic lipase is increased in T2DM; hence, the rate of sdLDL generation is increased [13]. These sdLDL particles are of smaller size that enables them to higher penetration rate into the arterial wall. After penetration, it may get easily oxidized and macrophage engulfs them to finally form the foam cell, which is a major contributor to atherosclerosis [14,15]. Figure 2 depicts the phenomenon in nutshell.

T2DM patients who are first-time diagnosed with the disease may have a high level of blood sugar level for a long period before the diagnosis. The HbA1c indicates a high blood glucose level for approximately the previous two to three months. Hence, in our study, the participants who showed high HbA1c might have an undefined period of hyperglycemia. And that might increase the level of hepatic lipase.

According to Hoogeveen et al., sdLDL-C is greater in diabetic individuals compared to nondiabetic patients and strongly correlates with an atherogenic lipid profile. They also reported that although patients with diabetes may have low cardiovascular risk as assessed by the level of LDL-C, sdLDL-C has the potential to predict the risk of coronary heart disease [16]. This is supported by another study by Koba et al. [17]. Not only in patients with T2DM but higher sdLDL-C may also be a risk factor for individuals with normal blood glucose levels [10]. Hence, it can be used for the prediction of risk for coronary artery disease [13]. Hence, an sdLDL-C report may be useful in patients diagnosed with T2DM with a "not at risk" category of a blood lipid profile to detect any hidden risk of heightened atherosclerosis.

In a low-risk population, a relationship has been found between LDL-C level and cardiovascular disease mortality in a large cohort with 10 years of follow-up. However, Abdullah et al. concluded that more research is needed to determine if lipid-modifying lifestyle changes and medical interventions improve cardiovascular disease outcomes in low-risk people with increased LDL-C [18]. The current study has found that although LDL-C may be within normal limits, the higher percentage of sdLDL-C may be a risk factor for diabetic patients. In addition, sdLDL-C is also associated with other risk markers like inflammation and thrombosis. Hence, sdLDL-C may be considered a promising biomarker [19]. However, the test is costly, and patients from developing countries like India may require framing guidelines only after further large-scale study. A cheaper alternative would be to measure apolipoprotein B (apoB) levels, which reflect sdLDL-C and score over LDL-C levels in patients with diabetes mellitus [20].

Research regarding cholesterol and related therapies is evolving. Statin therapy has been heavily marketed for cardiovascular prophylaxis since total cholesterol or LDL-C has been identified as the cause of atherosclerosis and cardiovascular disease. Though it is becoming increasingly clear that the processes are more nuanced and that statin therapy, particularly when used as primary prevention, is questionably beneficial [21,22].

There are both medicinal and non-medicinal approaches for control of the level of sdLDL-C. There is evidence and ongoing research about the beneficial effect of various nutritional supplements like almonds, avocados, dark chocolate, pistachios, and flaxseeds oil to control sdLDL-C. Exercise may also help in controlling the sdLDL-C level. The current medicinal treatment includes drugs like statins, fibrates, ezetimibe, niacin, and omega-3 fatty acids in various dosage forms. For a comprehensive review, readers may refer to the review by Jin et al. [23].

Limitations

This study is not a population-based study and was conducted with a sample taken from a tertiary care hospital as a convenience sample with inclusion and exclusion criteria. The duration for which the patients were suffering from T2DM was beyond our capability to measure. The conclusion drawn from the study conducted with a non-probability sample may not be generalized. Hence, the result of this study should be used with caution for clinical decisions.

## Conclusions

Newly diagnosed T2DM patients with blood lipids within an optimum or near-optimum level may have a higher percentage of sdLDL-C when compared with healthy controls. Hence, they may have a higher risk of atherosclerosis and cardiovascular disease risks despite having an LDL-C level that is considered normal or near-optimal. Hence, the sdLDL-C may be a hidden risk factor among patients otherwise of the "not at risk" category. However, the test for sdLDL-C is still costly for patients in developing countries. Clinicians dealing with patients with T2DM may consider the sdLDL-C level for patients who can afford the test for detecting a potential risk factor for cardiovascular diseases, which is not commonly practiced regularly.
